# Neutral Sodium Fluoride Gel Uptake of Newly Placed Nanodiamond-modified Glass Ionomers

**DOI:** 10.3290/j.ohpd.b871065

**Published:** 2020-12-14

**Authors:** Riaan Mulder, Nadia Mohamed, Madelaine Frazenburg

**Affiliations:** a Senior Lecturer, Department of Restorative Dentistry, Dental Faculty, University of the Western Cape, Parow, South Africa. Idea, hypothesis, experimental design, performed the experiments in partial fulfilment of requirements for a PhD degree, performed statistical evaluation, produced the images, wrote the manuscript.; b Professor, Department of Paediatric Dentistry, University of the Western Cape, Parow, South Africa. Evaluation of the manuscript, PhD supervisor of corresponding author.; c Senior Analyst, Department of Scanning Electron Microscopy (SEM), Stellenbosch University, Western Cape, Stellenbosch, South Africa. Performed SEM-EDS analysis, contributed substantially to the methodology design for SEM-EDS.

**Keywords:** elemental analysis, glass-ionomer cement, nanodiamonds, neutral sodium fluoride gel, SEM-EDS

## Abstract

**Purpose:**

Three commercial restorative glass-ionomer cements (GICs) were modified with 5% and 10 wt/wt% nanodiamond (ND) particles incorporated into the powder of the GICs. The aim of the study was to assess the percentage of surface fluoride increase on different materials, following 2% neutral sodium fluoride gel application (2% NSF).

**Materials and Methods:**

The commercial GICs were: FN, Fuji IX GP (GC); KU, Ketac Universal (3M Oral Care); and RSC: Riva Self Cure (SDI). Grade 1 (Plasmachem) nanodiamond was used. Six specimens of each material were prepared using precise powder:liquid ratios. After a 10-min setting time, the GICs were polished. The specimens were randomly divided into two groups: control (group A) and test (group B). The samples were blot dried and group B received the 2% NSF gel for 2 min. The excess was wiped off with gauze and both groups were analyzed with SEM-EDS.

**Results:**

Data analysis revealed that all the GICs and their respective ND modifications had a statistically significant surface fluoride percentage increase (p < 0.0001) on the GICs in group B. The addition of ND10% w/w to FN (p < 0.001) and RSC (p = 0.029) resulted in statistically significant increase of surface fluoride percentage. KU remained consistent with no statistically significant increase noted between ND-modified KU and KU in group A or B.

**Conclusion:**

The ability of GICs to absorb the 2% NSF gel immediately after finishing of the restoration statistically significantly increases the fluoride percentage of the surface layer.

Glass-ionomer cements (GICs) have anti-cariogenic properties due to the release of fluoride, which also influences biofilm metabolism.^1^,^30^

Fluoride plays a role in reducing the demineralisation of tooth structure.^26^ The effectiveness of GICs is therefore partly related to the fluoride release.^17^,^22^,^28^ Initially, after the placement of GICs, there is an increase in fluoride release over the first 24 h.^5^ Although GICs serve as fluoride reservoirs,^25^ they have the ability to replenish the fluoride potential from various fluoride sources. The replenishment of fluoride is essential to maintain a fluoride potential that can sustain a continuous release for biofilm modification^30^ and dental tissue mineralisation.^25^ GIC and resin-modified glass ionomer materials previously exposed to high fluoride concentrations have shown an enhanced fluoride release in deionised water.^4^,^6^

Nanodiamond (ND) particles have been identified by the authors as a potential material for the modification of GICs due to the presence of various functional groups. NDs are formed by the detonation of an explosive mixture of carbon-containing compounds, such as trinitrotoluene and hexogen (1,3,5-trinitro-1,3,5-triazinane) in a gaseous environment.^2^,^3^ The resultant ND has an sp3-carbon diamond core and a graphite outer layer presenting with an sp2-hybridized carbon.^20^ The grade 1 ND particles used in this study have oxygen-containing^27^ functional groups, e.g. hydroxyl groups, alkyls derived from sp3-carbon, carboxylic C=O groups and aromatic C=C groups.^12^ Due to these surface functional groups, NDs have been explored in various biomedical applications for many years^12^ and have been successfully studied for their application in medicine, such as for biosensing electrodes^32^ and ND films.^7^

The aim of this study was to determine the percentage of change in the surface fluoride of ND-modified GICs after 2% neutral sodium fluoride (2% NSF) gel application.

The null hypothesis was that the newly placed ND-modified GICs would have a higher surface fluoride percentage compared with the commercial GICs.

## MATERIALS AND METHODS

This in vitro study was approved by the ethics committee of the University of the Western Cape (BM/15/7/37).

### Study Design

Six disk-shaped specimens (3 mm in diameter and 1 mm thick) were constructed from each material. Three commercially available GICs – namely FN: Fuji IX GP hand-mix (GC; Tokyo, Japan, batch: 1503231); KU: Ketac Universal hand-mix; (3M Oral Care; St Paul, MN, USA, batch: 583514) and RSC: Riva Self Cure hand-mix (SDI; Bayswater, Australia, batch: 62657V) – were used in this study. The three GICs were additionally modified with 5% and 10% w/w nanodiamond particles (>98% nanodiamond particles, grade G01, Cat.Nr PL-D-G01; Plasmachem; Berlin, Germany) incorporated into the powder phase of the GICs as previously described.^14^ The GICs were mixed in keeping with the powder:liquid ratios as provided by the manufacturers.^13^ This was confirmed on a desktop chemical scale (Metler AE240 analytical balance; Columbus, OH, USA) by first dispensing the powder, followed by the liquid.

Six specimens per material were divided equally into two groups. Group A served as the control and group B was the test group, which was treated with 2% NSF gel.

The powder:liquid ratios of the GICs were weighed (Metler AE240 analytical balance) and mixed; then the GICs were applied into Teflon molds. Cellulose acetate sheets were placed on either surface of the mold with a glass slide over the cellulose acetate sheet. The GICs were allowed to bench set at 37ºC for 10 min in a temperature-controlled incubator (TOU50, MRC Laboratory instruments; Holon, Israel).^19^ The surface of all the specimens was smoothed with 2500-grit silicon carbide papers, followed by 4000-grit (3M Oral Care) under deionised water. By polishing, ± 100 µm of the surface was removed (accurate to the nearest micrometer). The application of the carbide paper simulated restoration polishing that would be performed by the clinician in the clinical setting.^11^ Lastly, the specimens were polished with diamond polishing paste (Eve Ernest Vetter; Keltern, Germany) and blot dried with filter paper (Whitman no.1, Cat No 1001125; GE Healthcare UK; Buckinghamshire, UK). The 2% NSF gel (Topex Neutral pH Sodium fluoride gel, Sultan Healthcare; Englewood, NJ, USA) was applied to the group B samples for 2 min. The 2% NSF gel was removed with gauze by wiping the samples twice from left to right while stabilising the sample with tweezers.

### SEM-EDS Microanalysis

Scanning electron microscopy (SEM) and Energy Dispersive X-Ray Spectrometry (EDS) microanalysis (SEM-EDS) were performed in group A (control) to establish the baseline percentage of fluoride and other elements as previously described.^14^ The process was repeated for group B. Surface elemental analysis was essential, since it has been shown that there are varying ions of GICs. The results from the SEM-EDS had to be representative of the GICs since the ‘glass particlesʼ have more ions than do the ʽmatrixʼ.^15^,^21^ The EDS spectra were collected from the central field of view from each specimen as previously described.^14^ Images were obtained at 200X and 500X magnification.

### Statistical Analysis

The SEM-EDS spectra from each material specimen were used to determine the average fluoride percentage for that specimen. No statistically significant outliers were present in the data as assessed by box plots and the studentised residuals. The residuals were studentised, since residuals cannot always be used (as it is unsure if an observation is an outlier or a variance is constant). Thus, the studentised residuals were subsequently calculated by dividing the residual by an estimate of its standard deviation. The standard deviation for each residual was computed with the observation excluded and the result was less than 3.0. The assessment of homogeneity of variances and covariances was not required, since the group sizes were equal. The data was assessed for normality using the Shapiro-Wilk test (p > 0.05) and homogeneity of variances using Levene’s test (p > 0.05). A one-way ANOVA and Tukey’s post-hoc analysis or the one-way Welch’s ANOVA with a Games-Howell post-hoc analysis was used for the analysis of the data, based on the Shapiro-Wilk and Levene’s tests.

## RESULTS

### Surface Fluoride Percentage of Three Commercial Materials before NaF Application (Group A)

The data from FN, KU and RSC had no statistically significant outliers, the data were normally distributed (Shapiro-Wilk test; p > 0.05) and homogeneity of variances (Levene’s test; p > 0.05) was present.

Table 1 presents the results of the one-way ANOVA and Tukey’s post-hoc analysis. The results revealed that the surface fluoride percentage of FN in group A had the highest fluoride percentage of 13.29% (±0.42), followed by RSC 11.71% (±0.16) and finally KU 11.35% (±0.35). The differences of surface fluoride percentages of the commercial materials in group A were statistically significant (p < 0.0001). Tukey’s post-hoc analysis (Table 2) revealed that the largest difference in the surface fluoride percentage was between FN – which had a statistically significantly larger percentage of surface fluoride – vs KU (p < 0.001) and RSC (p < 0.0001).

**Table 1:** Statistical significance of the change in the fluoride percentage from groups A to B

Material	F% before NaF (group A)	F% after NaF (group B)	Difference in F% (group B-group A)
FN	13.29 (0.42)	16.91 (1.07)	3.61 (0.67) ^
FN5%ND	11.94 (0.14)	16.39 (0.53)	4.44 (0.40) ^
FN10%ND	12.31 (0.69)	19.75 (0.07)	7.44 (0.74) ^
KU	11.35 (0.35)	20.32 (0.88)	8.96 (0.79) ^
KU5%ND	9.77 (0.62)	19.01 (0.37)	9.24 (0.53) ^
KU10%ND	10.18 (0.76)	19.62 (0.87)	9.44 (0.32) ^
RSC	11.71 (0.16)	16.32 (0.69)	4.6 (0.53) ^
RSC5%ND	10.85 (0.45)	14.85 (0.85)	4 (1.25) ^
RSC10%ND	11.09 (0.45)	16.88 (0.72)	5.79 (1.13) ^

Standard deviation represented in (). ^ indicates a statistically significant difference in the change of the fluoride percentage between groups A and B, p < 0.0001.

**Table 2:** Multiple comparison of the fluoride percentage difference between materials

Comparison	Statistical significance before NaF (group A)	Statistical significance after NaF (group B)
FN vs KU	p < 0.001*	p < 0.001* (KU vs FN)
FN vs RSC	p < 0.0001*	p = 0.856
FN vs FN5%ND	p < 0.001*	p = 0.087
FN vs FN10%ND	p = 0.002*	p < 0.001* (FN 10%ND vs FN)
FN 10%ND vs FN 5%ND	p = 0.228	p < 0.001*
KU vs RSC	p = 0.03* (RSC vs KU)	p < 0.001*
KU vs KU5%ND	p < 0.001*	p < 0.0001*
KU vs KU10%ND	p = 0.001*	p = 0.035*
KU5%ND vs KU10%ND	p = 0.214	p > 0.066 (KU10%ND vs KU5%ND)
RSC vs RSC5%ND	p < 0.001*	p < 0.001*
RSC10%ND vs RSC	p < 0.016*	p = 0.029*
RSC10%ND vs RSC5%ND	p = 0.337	p < 0.001*

* indicates a statistically significant difference in the fluoride percentage between the materials per group.

### Surface Fluoride Percentage of Three Commercial Materials after NaF Application (group B)

One-way Welch’s ANOVA was used for the analysis of group B for the three commercial materials, since Levene’s test showed no homogeneity of variances (p < 0.05; Table 2). The data for the three commercial materials in group B were normally distributed, as assessed by the Shapiro-Wilk test (p > 0.05). The differences between KU and FN as well as KU and RSC were statistically significant according to Welch’s F test (p < 0.0001). Table 1 shows that of the materials from group B, KU had the highest surface fluoride percentage after NaF treatment at 20.32 (±0.88)%, followed by FN 16.91 (±1.07)% and RSC 16.32 (±0.69)%. Games-Howell post-hoc analysis revealed that the difference between KU and RSC as well as KU and FN were statistically significant (p < 0.001). No statistically significant difference (p = 0.856) was noted between FN and RSC.

### Surface Fluoride Percentage of the ND-modified GICs

Data analysis revealed that the surface fluoride percentage difference for the three commercial GICs as well as the 5%ND and 10%ND modifications between group A and group B was statistically significant (p < 0.0001), with a surface fluoride increase on the surface of the GICs (Table 1).

In group B, after the application of the 2% NSF gel, the addition of 10% w/w ND to FN resulted in FN10%ND having a statistically significant (p < 0.001) increase of surface fluoride percentage for FN. Additionally, RSC10%ND had a statistically significantly greater surface fluoride percentage than did RSC (p = 0.029). The results presented in Table 1 show that the modification of KU with both 5% and 10%ND showed no statistically significant increase of surface fluoride of the ND-modified KU materials when compared with the commercial KU material in groups A and B. Therefore, not only was there no statistically significant increase of surface fluoride, but the total fluoride percentage was lower than on the unmodified samples.

Table 1 demonstrates the increase of surface fluoride percentage between groups A and B with FN by 3.61 (0.67)%, FN5%ND by 4.44 (0.40)% and FN10%ND by 7.44 (0.74)%. The surface fluoride percentage in group B was statistically significantly different between FN10%ND and FN (p < 0.001), as well as between FN10%ND and FN5%ND (p < 0.001). However, the difference between FN and FN5%ND (p = 0.087), was not statistically significant (Table 2).

Table 1 presents surface fluoride percentage increases between groups A and B for KU by 8.96 (±0.79)%, KU5%ND by 9.24 (±0.53)% and KU10%ND by 9.44 (±0.32)%. The KU surface fluoride percentage increase of group B was statistically significant compared to KU10%ND (p = 0.035), followed by KU5%ND (p < 0.0001) (Table 2).

For RSC, the surface fluoride percentage increased for RSC by 4.6% (±0.53), RSC5%ND by 4.00% (±1.25) and RSC10%ND by 5.79 (±1.13)% (p < 0.001). RSC10%ND resulted in a statistically significant increase in group B, which was also greater than the fluoride increase of RSC (p = 0.029), followed by RSC5%ND (p < 0.001) (Table 2).

## DISCUSSION

Where the increased surface fluoride percentage of the commercial GICs was concerned, the null hypothesis was accepted for FN10%ND and RSC10%ND and rejected for the other ND-modified GICs.

**Fig 1:**
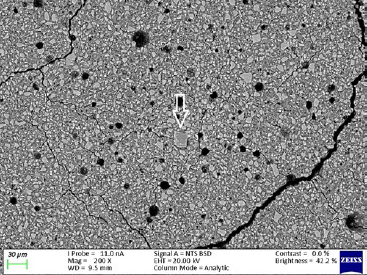
Ketac Universal from group B at 200X magnification.

**Fig 2:**
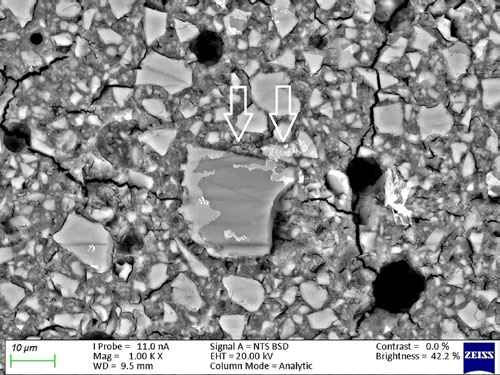
Ketac Universal from group B at 500X magnification.

**Fig 3:**
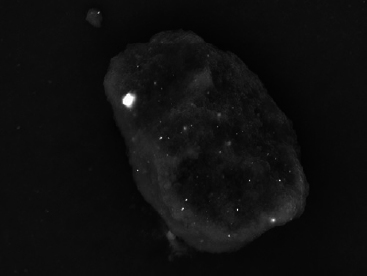
4-nm nanodiamond PL-D-G01 particles.

**Fig 4:**
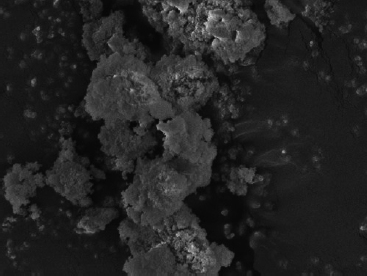
5-15 nm nanodiamond PL-D-G01 aggregate.

**Fig 5:**
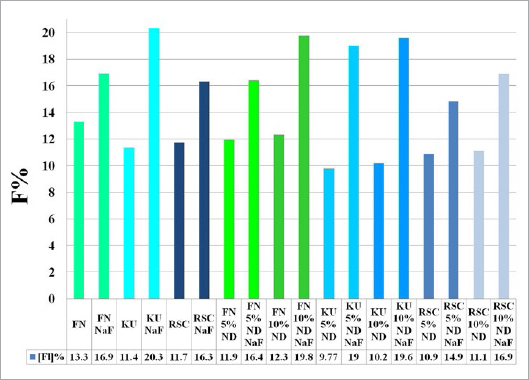
Percentage of fluoride before and after NaF treatment (from groups A to B).

The ND modifications of the commercial GICs resulted in a decrease in fluoride percentage. This was due to the fact that ND particles have a porous structure and the free fluoride released during the acid-base reaction was absorbed into the ND particles. The statistically significant increases in the percentage of fluoride in the ND-modified GICs were due to the porosity of the ND particles and the multiple functional groups on their surface, which attract fluoride ions from the 2% NSF gel. This in vitro study illustrated the statistically significant increase of surface fluoride percentage following 2% NSF gel application immediately after finishing the GICs. As noted in a SEM study of GICs using 1.1% NSF gel, the use of a neutral sodium fluoride gel did not change the surface roughness of Fuji IX,^18^ and no damage to the GIC surfaces was noted.^5^ This was confirmed in the present in vitro study (Fig 1). Upon further investigation at higher magnification, it was clear how the 2% NSF gel interacted with the surface of the matrix and filler particles. A well-covered GIC surface was noted with a thin film of dried 2% NSF gel, which flaked off of the filler particles (Fig 2). The addition of NDs to GICs may therefore be more important considering that the fluoride ion is able to interact and retain a greater percentage of fluoride in FN10%ND and RSC10%ND. Kealey et al^7^illustrated that these PL-ND-G01 ND particles exposed to fluoride did not show any physical changes. The incorporation of the ND particles in the GIC powder was easily achieved, since ND particles have a coherent scattering region size of 4 nm (Fig 3) with an aggregate size of 5-15 nm^31^ (Fig 4). The interaction of fluoride with ND was considered favourable, since fluoride has an affinity for the graphite components of the ND particles^9^ and also interacts with the van der Waals forces. Therefore, the GICs modified with NDs continuously recharge upon fluoride exposure, retaining the recharge functionality of the commercial GICs. The long-term fluoride release from GICs is mitigated through the matured matrix.^10^,^24^ Although the commercial GICs had a significant increase in fluoride on the surface, the ND-modified GICs (with the exception of KU) illustrated a statistically significant increase in fluoride percentage. With this increased fluoride percentage demonstrated by all the GICs and the ND-modified GICs, the gradient potential of fluoride available for release to the tooth structure and the oral environment is an important clinical application. The varying combination of ions (Table 3) influences the percentage of fluoride that can interact with the ions in the GICs. The ND particles with their functional group also interact with the aluminium, lanthanum, silica and strontium of the commercial GICs (Table 3). These elements interact during the acid-base reaction and occupy many bonding sites on the NDs. This is illustrated with the first drop in the fluoride percentage of ND-modified GICs compared to the commercial GICs in group A (Table 1, Fig 5). Ion release of aluminium, silica, sodium and strontium into de-ionised water from the ND-modified GICs illustrated an increased release of ions to their respective commercial materials.^14^ In a study where fluoride release was determined with both TISAB III and TISAB IV analysis, the ND-modified GICs presented an increased fluoride release compared to their respective commercial materials.^15^

**Table 3:** Elemental analysis of the surface elemental percentage of the commercial GICs of group A

Material	Aluminium^14^	Calcium	Fluoride	Lanthanum	Phosphate	Silica^14^	Sodium^14^	Strontium^14^
FN	11.181	0.223	13.294	0.000	3.104	10.183	0.906	3.854
KU	8.151	0.172	11.357	2.586	2.494	10.464	1.813	2.782
RSC	9.793	0.096	11.719	0.000	2.349	9.911	1.266	3.048

When comparing the ND-modification results between groups A and B with the commercial materials in Table 1, it becomes apparent that there is an ND threshold. The FN, KU and RSC modified with ND-5% had a lower fluoride percentage after 2% NSF exposure compared to their respective ND-10% modifications, where it was clearly sufficient to increase the fluoride percentage. With regard to RSC vs RSC5%ND, the ND-modified material was below the sufficiency threshold to increase the fluoride percentage to a lager extent than this commercial material. The difference in fluoride percentage of KU and its ND-modifications between group A and B was the largest compared to the other two commercial GICs (Table 1). KU was the only GIC that contained lanthanum. Lanthanum can have a large affinity for fluoride.^23^ In this in vitro study, the GICs completed the initial acid-base reaction; when the 2% NSF gel interacts at a pH ≤ 7.5 with lanthanum hydroxide, an exceptionally high fluoride absorption value of up to 242,200 ppm can occur with the lanthanum.^21^ Fluoride may exist in the form of free or complexed ions after the acid-base reaction of the maturing GICs is complete. The fluoride binds to the lanthanum hydroxide by exchanging fluoride for hydroxides on the lanthanum molecule.^16^ The increase of hydroxides raises the pH. At a pH > 8.7, the ability of the lanthanum hydroxide to adsorb fluoride sharply decreases, since the sites on the lanthanum are already predominantly occupied by fluoride. However, with its negative charge, fluoride does have a strong interaction with aluminium and calcium ions as well, linked in the polyalkenoate chains of the silica matrix of the mature restoration. This interaction is why fluoride has a slow and continuous movement through the matured matrix.^29^ Application of 2% NSF gel after finishing the GICs is advantageous. Even after the GICs have matured, the fluoride will be able to substitute the available terminal hydrogen (H) or hydroxyl (OH) species on the surface of the ND particles added to the GICs.^8^

### Study Limitations

The effects of GIC modification with NDs on physical properties, ion release into de-ionised water^14^and chemical interaction with tooth structure^15^ have been studied elsewhere, but were beyond the scope of this article.

It would be clinically valuable to evaluate the release and re-release of 2% NSF gel from the ND-modified GICs. Since the graphite components have an affinity for fluoride, the release and re-release results as well as the change in the pH gel over the 2-min interaction with the ND-modified GICs could provide insight to release and re-release results.

## CONCLUSION

In order for the tooth and the restoration to benefit from the fluoride ions, it is advisable to apply 2% NSF gel directly after finishing GIC restorations. The ND-modified GICs, namely FN10%ND and RSC10%ND, showed a significantly larger percentage of surface fluoride than did the FN and RSC commercial materials. All the ND-modified GICs (besides RSC5%ND) had a larger surface fluoride percentage increase than their respective commercial materials. ND particle incorporation into GICs therefore improves the surface fluoride percentage of FN and RSC in 10% w/w integration in GIC powder.

## References

[ref1] Chau N, Pandit S, Cai J, Lee M, Jeon J (2015). Relationship between fluoride release rate and anti-cariogenic biofilm activity of glass ionomer cements. Dent Mater.

[ref2] Danilenko V (2004). On the history of the discovery of nanodiamond synthesis. Solid State Phys.

[ref3] Dolmatov V (2001). Detonation synthesis ultradispersed diamonds: properties and applications. Russ Chem Rev.

[ref4] El Mallakh B, Sarkar N (1990). Fluoride release from glass-ionomer cements in de-ionized water and artificial saliva. Dent Mater.

[ref5] El-Badrawy W, McComb D (1998). Effect of home-use fluoride gels on resin-modified glass ionomer cements. Oper Dent.

[ref6] Forsten L (1990). Short- and long-term fluoride release from glass ionomers and other fluoride-containing filling materials in vitro. Eur J Oral Sci.

[ref7] Kealey C, Klapötke T, McComb D, Robertson M, Winfield J (2001). Fluorination of polycrystalline diamond films and powders. An investigation using FTIR spectroscopy, SEM, energy-filtered TEM, XPS and fluorine-18 radiotracer methods. J Mater Chem.

[ref8] Kaur R, Badea I (2013). Nanodiamonds as novel nanomaterials for biomedical applications: drug delivery and imaging systems. Int J Nanomedicine.

[ref9] Kita Y, Watanabe N, Fujii Y (1979). Chemical composition and crystal structure of graphite fluoride. J Am Chem Soc.

[ref10] Matsuya S, Maeda T, Ohta M (1996). IR and NMR analyses of hardening and maturation of glass-ionomer cement. J Dent Res.

[ref11] Menne-Happ U, Ilie N (2013). Effect of heat application on the mechanical behaviour of glass ionomer cements. Clin Oral Invest.

[ref12] Mochalin V, Shenderova O, Ho D, Gogotsi Y (2011). The properties and applications of nanodiamonds. Nat Nanotechnol.

[ref13] Mulder R (2018). Variation in the dispersions of powder liquid ratios in hand-mix glass ionomers. Open Dent J.

[ref14] Mulder R, Anderson-Small C (2019). Ion release of chitosan and nanodiamond modified glass ionomer restorative cements. Clin Cosmet Investig Dent.

[ref15] Mulder R (2019). An in vitro study of the properties of GICs with bioactive biomaterial modification. University of the Western Cape.

[ref16] Na C, Park H (2010). Defluoridation from aqueous solution by lanthanum hydroxide. J Hazard Mater.

[ref17] Nakajo K, Imazato S, Takahashi Y, Kiba W, Ebisu S, Takahashi N (2009). Fluoride released from glass-ionomer cement is responsible to inhibit the acid production of caries-related oral streptococci. Dent Mater.

[ref18] Ozdemir-Ozenen D, Sungurtekin E, Issever H, Sandalli N (2003). Surface roughness of fluoride-releasing restorative materials after topical fluoride application. Eur J Paediatr Dent.

[ref19] Pawluk K, Booth S, Coleman N, Nicholson J (2008). The kinetics of fluoride uptake from aqueous solutions by immature glass-ionomer dental cements. Dent Forum.

[ref20] Roy S, Mitra K, Desai C, Petrova R, Mitra S (2013). Detonation nanodiamonds and carbon nanotubes as reinforcements in epoxy composites—a comparative study. J Nanotechnol Eng Med.

[ref21] Selig W (1970). Potentiometric micro- and semimicro-determination of fluorine in organic compounds. Fresenius Z Anal Chem.

[ref22] Trairatvorakul C, Itsaraviriyakul S, Wiboonchan W (2010). Effect of glass-ionomer cement on the progression of proximal caries. J Dent Res.

[ref23] Vardhan CMV, Srimurali M (2016). Removal of fluoride from water using a novel sorbent lanthanum-impregnated bauxite. Springerplus.

[ref24] Verbeeck R, De Maeyer E, Marks L, De Moor R, De Witte A, Trimpeneers L (1998). Fluoride release process of (resin-modified) glass-ionomer cements versus (polyacid-modified) composite resins. Biomater.

[ref25] Walls A (1986). Glass polyalkenoate (glass-ionomer) cements: a review. J Dent.

[ref26] Watson T, Atmeh A, Sajini S, Cook R, Festy F (2014). Present and future of glass-ionomers and calcium-silicate cements as bioactive materials in dentistry: Biophotonics-based interfacial analyses in health and disease. Dent Mater.

[ref27] Wehling J, Dringen R, Zare R, Maas M, Rezwan K (2014). Bactericidal activity of partially oxidized nanodiamonds. ACS Nano.

[ref28] Wiegand A, Buchalla W, Attin T (2007). Review on fluoride-releasing restorative materials—Fluoride release and uptake characteristics, antibacterial activity and influence on caries formation. Dent Mater.

[ref29] Wilson A, Groffman D, Kuhn A (1985). The release of fluoride and other chemical species from a glass-ionomer cement. Biomater.

[ref30] Xie D, Brantley W, Culbertson B, Wang G (2000). Mechanical properties and microstructures of glass-ionomer cements. Dent Mater.

[ref31] Yakovlev R, Osipova A, Solomatin A, Kulakova I, Murav’eva G, Avramenko N (2015). An approach to unification of the physicochemical properties of commercial detonation nanodiamonds. Russ J Gen Chem.

[ref32] Zhang W, Patel K, Schexnider A, Banu S, Radadia A (2014). Nanostructuring of biosensing electrodes with nanodiamonds for antibody immobilization. ACS Nano.

